# *SPTSSA* Is a Prognostic Marker for Glioblastoma Associated with Tumor-Infiltrating Immune Cells and Oxidative Stress

**DOI:** 10.1155/2022/6711085

**Published:** 2022-08-24

**Authors:** Ziheng Wang, Xinqi Ge, Jinlong Shi, Bing Lu, Xiaojin Zhang, Jianfei Huang

**Affiliations:** ^1^Department of Clinical Biobank & Institute of Oncology, Affiliated Hospital of Nantong University, Nantong, China 226000; ^2^Department of Neurosurgery, Affiliated Hospital of Nantong University, China 226000

## Abstract

**Background:**

*SPTSSA* encodes the small subunit A of serine palmitoyltransferase. It catalyzes the formation of sphingoid long-chain base backbone of sphingolipids. Its role in glioma prognosis and tumor-infiltrating immune cells remains unclear.

**Methods:**

We analyzed *SPTSSA* expression and association with clinical prognosis using GEPIA and CGGA database. Then, GSEA was performed to identify relevant biological functions of *SPTSSA*. The correlations between *SPTSSA* expression and tumor immune infiltrates were investigated using CIBERSORT and TIMER. Finally, IHC and IF were performed to confirm the value of prognosis and the correlation with immune infiltration.

**Results:**

*SPTSSA* expression was significantly upregulated in diffuse glioma compared to normal tissues and associated with poor survival in GEPIA and CGGA database. Then, we identified biological processes and signaling pathways associated with *SPTSSA* expression. The result showed that *SPTSSA* enriched in the GO term like oxidative stress. Finally, we showed that *SPTSSA* expression was significantly associated with tumor-infiltrating immune cells and overall survival via IHC.

**Conclusion:**

These findings suggest that *SPTSSA* expression might be used as a prognostic biomarker for glioma and potential target for novel glioma therapy.

## 1. Introduction

In adults, in the central nervous system, the glioblastoma multiforme (GBM) is known to be the most prevalent form of malignancy. Representing almost 15% of all brain tumors, it has an incidence of 3.4 per 100,000 [1–3]. Eighty percent of GBMs is primary (de novo) GBMs and mainly occurs in older patients; the remaining GBMs are secondary GBMs derived from lower-grade astrocytoma or oligodedrogliomas and mainly occur in younger patients. GBM is an aggressive neoplasm; if untreated, patients have a median survival of 3 months [4]. The current standard treatments include surgical resection, chemotherapy with temozolomide, and radiotherapy [5]. With a median survival level of 12-14 months, the prognosis of GBM remains poor despite the advances in radiotherapy and surgery. Less than 5% of patients survive longer than 5 years after diagnosis [6–8]. Novel GBM treatments with improved clinical outcomes are urgently needed.

Cancer immunotherapy takes advantage of the body's own immune system to eradicate tumor cells [9, 10]. Current GBM immunotherapy approaches include checkpoint inhibitor treatment, adoptive cell therapy, dendritic-cell-based therapy, and peptide vaccination [11, 12]. Because the immune system plays a key role in the formation and establishment of tumors, a deep understanding of tumor microenvironment is essential to elucidate tumor-immune interactions and develop effective immunotherapy for GBM. Previous studies suggest that both tumor-associated macrophages (TAMs) and tumor-infiltrating neutrophils (TINs) could affect the treatment outcome and overall survival in GBM [13–16]. However, comprehensive analysis of various immune cell subtypes of GBM is lacking.

With the rapid development of various techniques for gene expression analysis and accumulation of large gene expression databases on clinical samples, bioinformatics analysis plays a significant role in screening and identification of candidate biomarkers for various diseases including cancers [17–19]. Bioinformatics not only provides data for identification of functionally differentially expressed genes (DEGs) for cancer diagnosis and prognosis but can also infer the percentage of tumor-infiltrating immune cells from gene expression profiles [20, 21]. Finally, IHC result confirmed that *SPTSSA* was a novel biomarker associated with the infiltrating immune cells.

In the current study, we used bioinformatics analysis on GEPIA and CGGA databases and identified *SPTSSA* expression correlating with the prognosis of glioma patients. We further determined the correlation of *SPTSSA* expression with tumor-infiltrating immune cells using CIBERSORT, TIMER, and IHC. Our data provide rationale for future clinical and experimental studies of *SPTSSA* in GBM.

## 2. Material and Methods

### 2.1. GEPIA Dataset Analysis

We used Gene Expression Profiling Interactive Analysis (GEPIA) (http://gepia.cancer-pku.cn/), an interactive web server to identify cancer types that showed differential expression of *SPTSSA* gene between cancerous and normal tissues. Among cancer types that demonstrated differential expression of *SPTSSA* gene, we analyzed the association of *SPTSSA* expression and overall survival.

### 2.2. Clinical Information and the CGGA mRNA Matrix

In this study, the glioma samples obtained from the CGGA network (http://www.cgga.org.cn) numbering 1018 were included. The respective clinicopathological information and the informed consent for all these samples were obtained. The institutional review board of the Tiantan Hospital approved this study. To ascertain the differences in the *SPTSSA* expression and the survival value, an analysis was conducted initially. Besides, the mRNAseq_325 (Illumina HiSeq 2000 or 2500) and the mRNAseq_693 (Platform: Illumina HiSeq) datasets were also downloaded for further investigations. From the total number of 1,018 glioma samples comprising the two datasets, 693 samples were from mRNAseq_693, and 325 samples were from the mRNAseq_325. For batching and normalizing the two mRNA matrices, the limma and Sva packages were utilized.  Table 1 enumerates the 749 completed clinical information contained in the clinicopathological characteristics of the patients received from the CGGA databases. The R software was used to conduct the gene expression and the survival analyses (version 3.6.2).

### 2.3. Gene Set Enrichment Analysis (GSEA)

To determine the statistical significance of a previously outlined set of genes and the presence of consistent differences concerning two biological states, researchers utilize a computational method like GSEA [22, 23]. In this research, GSEA produced a preliminary list that classified the genes based on their association with the *SPTSSA* expression. Moreover, it elaborates on the remarkable differences between the survival of low- and high-*SPTSSA* groups.

In every analysis, gene set permutations were performed repetitively for 1000 times. We created a phenotype label based on the *SPTSSA*'s expression level. Furthermore, we used the normalized enrichment score (NES) and nominal *p* value to categorize the enriched pathways in every phenotype [24]. Significantly enriched gene sets occurred at a discovery rate of |*NES*| > 1 and (FDR) < 0.05.

### 2.4. Tumor-Infiltrating Immune Cell Analysis Using CIBERSORT

CIBERSORT is a gene expression-based analytical tool for characterizing immune cell composition (http://cibersort.stanford.edu). Using CIBERSORT, we calculated the percentage of infiltrating immune cells in glioma tissues. Wilcox test was used to analyze the difference between *SPTSSA* high- and low-expression groups. R language survival package was used to determine the relationship between infiltrating immune cells and overall survival.

### 2.5. TIMER Database Analysis

Tumor Immune Estimation Resource (TIMER, http://cistrome.shinyapps.io/timer/) is a web server for comprehensive analysis of tumor-infiltrating immune cells. Using TIMER database, we further validated the correlation between *SPTSSA* expression and tumor-infiltrating immune cells.

### 2.6. Sample Collection

The *SPTSSA* expression analysis utilized IHC to assess the glioma tissues of 35 patients on paraffin-embedded, formalin-fixed slides. These samples were collected from the Affiliated Hospital of Nantong University from 2004 to 2014. Using these tissues, the TMA was constructed through the Tissue Microarray System (Quick-Ray, UT06, Unitma, Seoul, South Korea) based on the approach mentioned previously. The clinicopathological information obtained included the differentiation grade, histological type, age, and sex. All patients in the study signed and issued a written informed consent. The Affiliated Hospital of Nantong University's Human Research Ethics Committee approved the study protocol (2018-K020).

### 2.7. Construction of the Tissue Microarray (TMA)

From the tissue areas containing >50% tumor, the pertinent regions were selected for the TMA construction from each block of the glioma tissue. Using the MTA-1 Manual Tissue Arrayer (Beecher Instruments, Sun Prairie, WI, USA), representative tumor cores measuring around 1 mm and two/three in number were transferred from the glioma tissue blocks to the recipient TMA blocks in each case. In this manner, the TMAs were constructed.

### 2.8. Preparation of Monoclonal Antibody against *SPTSSA*

Mab (monoclonal antibody) was prepared from female SPF (specific pathogen free) mice which firstly received 60 *μ*g polypeptide (3.0 mg/mL) subcutaneous injections. Then, four mice received either four subcutaneous injection polypeptide. Indirect ELISA was performed to analyze the titer of IgG antibody. No. 4 mouse was chosen to perform cell fusion.

### 2.9. Immunohistochemistry (IHC)

To quench the endogenous peroxidase, the TMA sections were incubated for 15 minutes with methanol and 3% H_2_O_2_ after being deparaffinized. By heating the sections in sodium citrate buffer (10 mmol/L, pH 6.0) for 3 minutes in a pressure cooker, the antigen was retrieved. Subsequently, for one hour, with the primary goat anti-*SPTSSA* antibody-diluted bovine serum albumin, the tissue sections were incubated. The phosphate-buffered saline was used to wash the sections before being incubated for 15 minutes with horseradish peroxidase-conjugated donkey anti-goat antibody (Abcam) and washed further. Prior to the light counterstaining with hematoxylin, the sections were incubated for 15 minutes with diaminobenzidine solution (Kem-En-Tec Diagnostics, Taastrup, Denmark) to develop the color.

### 2.10. Immunofluorescence Staining

The glioma samples were cut in thin sections of 3 *μ*m for immunostaining. Using the Fluorescence Kit (NEL 797001KT; PerkinElmer) and the Opal 8-color Fluorophore TSA, the multiplex immunofluorescence (IF) was done for CD83/CD56/CD20/CD68/CD66b/CD8/CD4/CD3 on the 3 *μ*m formalin-fixed glioma tissue sections. Since combining four or more antibodies using IHC was challenging technically, instead of IHC, we performed the multiplexed IF.

### 2.11. Multispectral Analysis and Imaging

With an interactive image segmentation system, the Vectra 3 automated quantitative pathology imaging system, the image analysis, and acquisition were performed on the glioma slides. According to the intensity and the staining pattern on each selected image, the pathologist decided the fluorescent intensity count cutoff value for positivity for each marker of interest (CD83/CD56/CD20/CD68/CD66b/CD8/CD4/CD3). The extragerminal center area and the germinal center area were included for each image of the normal glioma tissue. The manual tissue segmentation function of the software was utilized for differentiating the two areas and analyzed independently.

### 2.12. Survival Analysis

Using *SPTSSA* medium expression level, glioma samples were stratified into high-*SPTSSA* expression and low-*SPTSSA* expression groups. Kaplan-Meier survival analysis was used to estimate the survival distributions. Evaluating the statistical significance required the log-rank test between stratified survival groups through the GraphPad Prism package. Then, we filtered the survival and gene expression data using Cox regression analyses at *p* < 0.05.

## 3. Results

### 3.1. *SPTSSA* Was Significantly Upregulated in Diffuse Glioma Compared to Normal Tissues and Associated with Poor Survival

**Figure 1:**
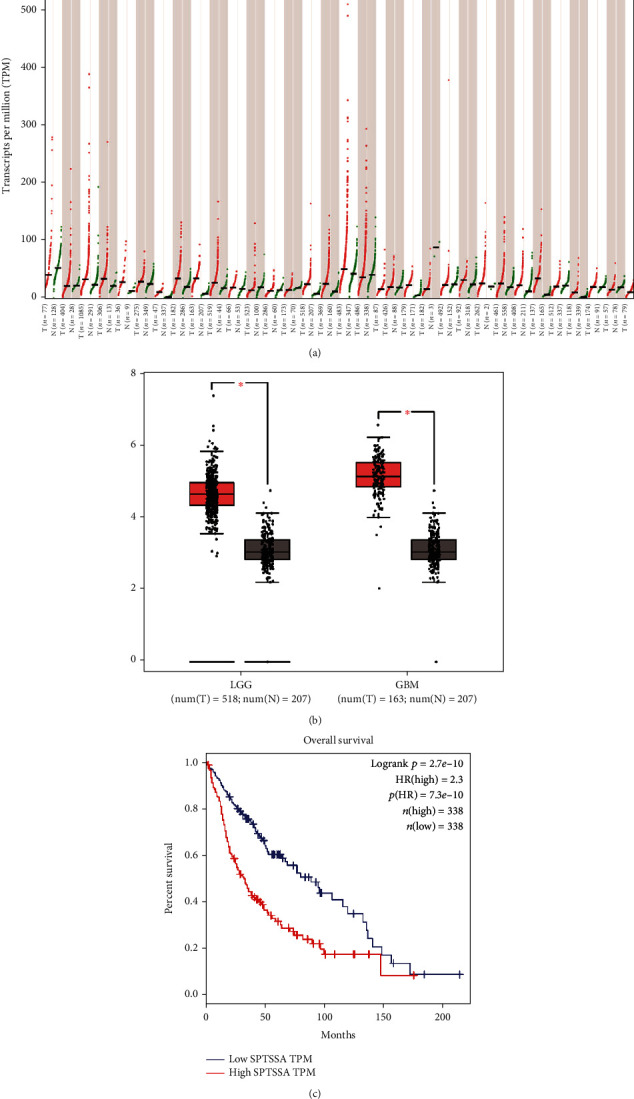
(a) Overview of expression of all cancers and normal tissues in GEPIA database. (b) Differences of SPTSSA in normal cells, low-grade glioma, and glioblastoma. (c) Grouped by median, high expression of SPTSSA was associated with poor survival.

We searched Gene Expression Profiling Interactive Analysis (GEPIA) database for *SPTSSA* expression in various tumor types. TCGA (http://tcga-data.nci.nih.gov/tcga/) and GTEx (http://commonfund.nih.gov/GTEx/) datasets from 33 tumor types were retrieved. The analysis of gene expression profile across all tumor samples and paired normal tissues indicated that seven tumor types (DLBC, GBM, LGG, LIHC, PAAD, TGCT, and THYM) showed significant higher *SPTSSA* expression in tumor tissues than in normal tissues (Figure 1(a)). Among these seven tumor types, we separated tumor cases into high-*SPTSSA* expression cases (>median expression level) and low-*SPTSSA* expression cases (≤median expression level) (Figure 1(b)). Log rank survival analysis indicated that high-*SPTSSA* expression in GBM and LGG was associated with poor survival (HR = 2.3, *p*(HR) = 7.3*e* − 10, Logrank *p* = 2.7*e* − 10) (Figure 1(c)).

### 3.2. Correlation of *SPTSSA* Expression with Clinical Characteristics

**Figure 2:**
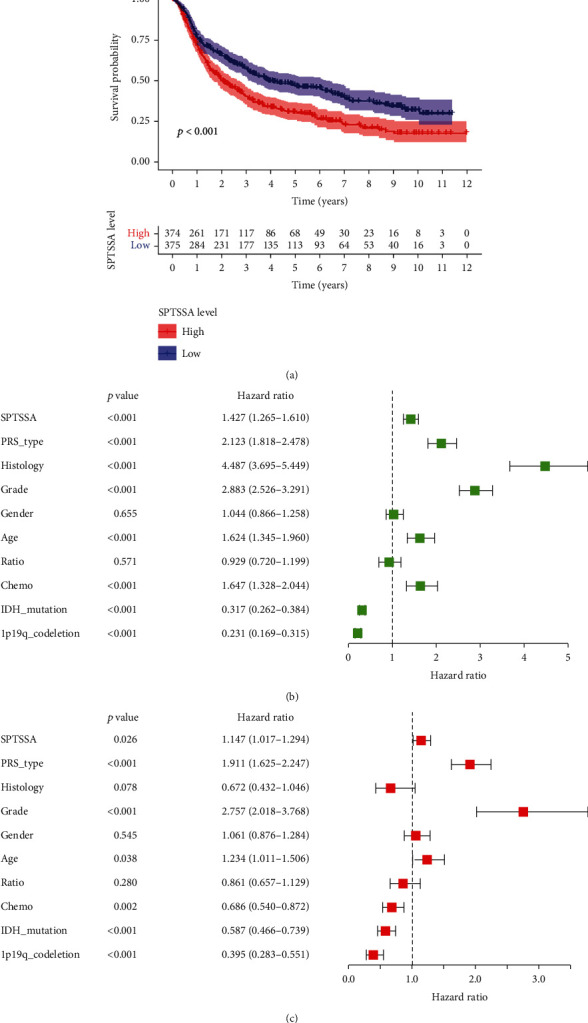
(a) Using CGGA database, grouped in median, high expression of SPTSSA predicts poor prognosis. (b, c) Univariate and multivariate Cox analyses indicated that SPTSSA was an independent predictor for OS.

To confirm our observation, we next analyzed *SPTSSA* expression using CGGA database. Log-rank test analysis showed that high-*SPTSSA* expression was significantly associated with poor survival (*p* < 0.001) (Figure 2(a)). In univariate analysis, PRS-type, histology, grade, age, chemotherapy, IDH-mutation, 1p19q-codeketion, and *SPTSSA* expression were all significantly associated with survival (Figure 2(b)). In multivariate analysis, PRS-type, grade, IDH-mutation, 1p19q-codeletion, and *SPTSSA* remained significantly associated with survival (Figure 2(c)). Moreover, a nomogram was carried out to investigate individualized survival probability (Supplementary Figure 1A), and calibration curve was carried out to demonstrate the accuracy of the nomogram in predicting prognosis at different time points (Supplementary Figure 1B). The cox analysis between SPTSSA and OS, PFI, DSS, and DFI was also carried out (Supplementary Figure 2A-2D). The results showed that SPTSSA correlated positively with OS, PFI, and DSS based on pan-cancer analysis. All the results indicate that SPTSSA could serve as an independent prognostic predictor for glioma.

### 3.3. Multifactorial Integrated Survival Analysis in CGGA Database

**Figure 3:**
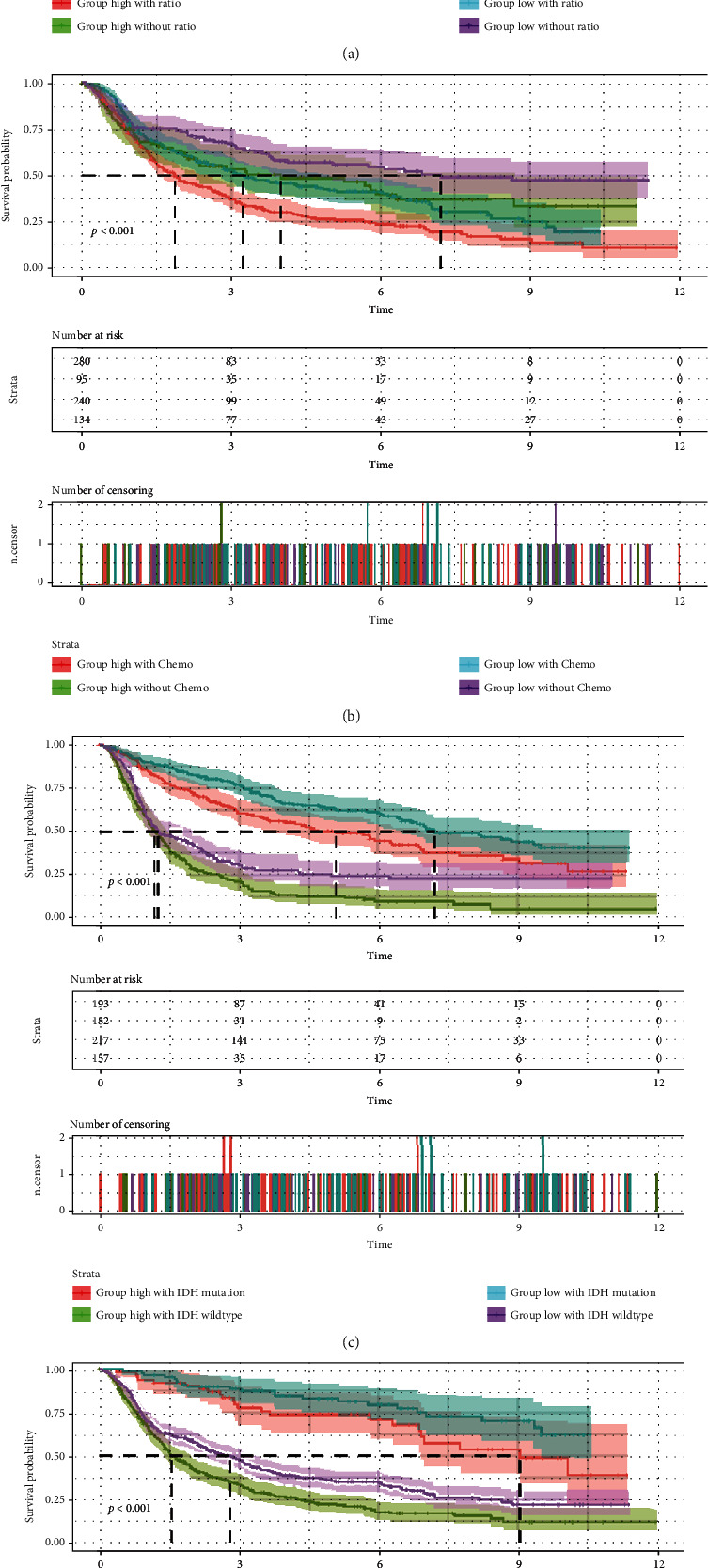
Survival analysis of CGGA patients to SPTSSA expression compared with (a) radiotherapy, (b) chemotherapy, (c) IDH mutation, and (d) 1p19q status.

The radiotherapy (Figure 3(a)), chemotherapy (Figure 3(b)), IDH1 genotypes (Figure 3(c)), and 1p19q status (Figure 3(d)) were added as variables in multifactorial analysis to further investigate the clinical value of *SPTSSA*. The correlation between the expression of *SPTSSA* and the survival rate with chemotherapy was analyzed subsequently. The poorest outcome was noticed in the case of the highest *SPTSSA* expression with chemotherapy (Figure 3(b), cherry), whereas the expression of *SPTSSA* without chemotherapy (Figure 3(b), purple) indicated the lowest. The role of the corresponding radiotherapy (*p* < 0.0001) *SPTSSA* as an important indicator was evidenced by the higher expression of *SPTSSA* (Figure 3(a), cherry and green) in the IDH1-mutant groups (Figure 3(a), purple and blue) revealing poor survival. The patients with high expression of *SPTSSA* (Figure 3(d), green) and noncodel 1p19q finally indicated the worst prognosis.

### 3.4. Correlations between *SPTSSA* and Immunotherapy, Immunotherapy Response Prediction

Cancer immunotherapy is radically transforming cancer [25], and the use of immunotherapy in cancer treatment is on the rise [26]. Several studies have shown that immunotherapy and targeted therapy are effective in melanomas [27]. Thus, it is necessary to investigate the relationship between *SPTSSA* and immunotherapy and immunotherapy response prediction. In our study, we downloaded 109 samples from GSE91061, and the results showed that *SPTSSA* expression level has a positive correlation with anti-PD-1/CTLA-4 therapy both in LGG and GBM (Supplementary Figure 3A). And immunotherapy response prediction of AUC is 0.688 which reveals that *SPTSSA* can predict immunotherapy response of glioma patients (Supplementary Figure 3B).

Furthermore, we investigated the relationship between SPTSSA and immune suppressive factors, immune promoting factors, MHC factors, chemokine, and receptors (Supplementary Figure 3C). The results showed that SPTSSA has a positive relationship with most immune suppressive factors, immune promoting factors, MHC factors, chemokine, and receptors which indicate that SPTSSA could have a good immune therapy towards glioma patients.

### 3.5. Connections between *SPTSSA* and Genomic Alteration

The majority of cancers harbor at least one genomic alteration that could lead to potential treatment options, with 84% showing at least one. And tailored medicine is often based on specific genetic alterations that improve treatment outcomes [28]. Thus, exploring the relationship between *SPTSSA* and genomic alteration seems to be necessary. In our study, we found that SPTSSA is positively correlated with *CALN1* in GBM, while no positive correlated genomic alteration found in LGG. And in GBM, the gain of *SPTSSA* genomic alteration located in 17q13.2, while the loss of *SPTSSA* genomic alteration located in 14q13.1 and 14q24.2 (Supplementary Figure 4A-4B).

### 3.6. Gene Set Enrichment Analysis (GSEA)

**Figure 4:**
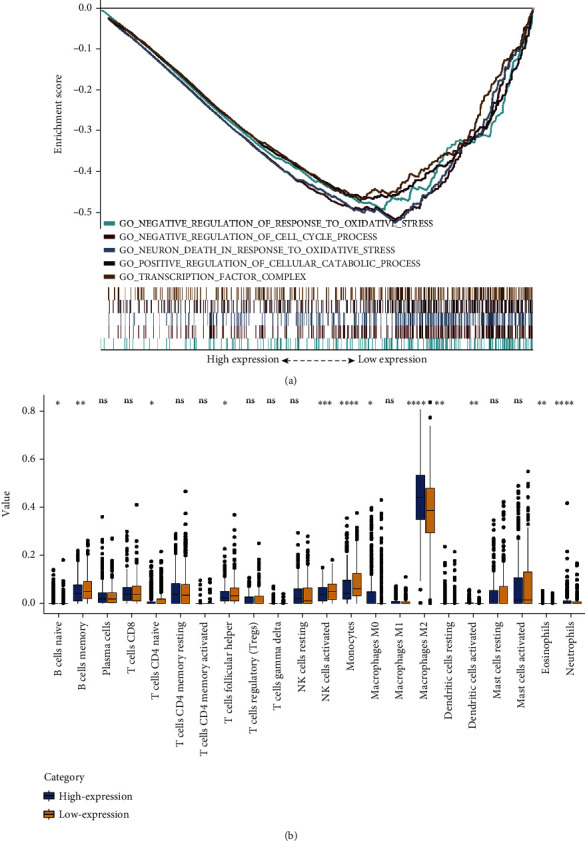
(a) Enrichment plots from the Gene Set Enrichment Analysis. (b) The proportions of 22 tumor-infiltrating immune cells in high-SPTSSA and low-SPTSSA expression group.

To distinguish the differentially activated signaling pathways in GBM, we performed the GSEA between low- and high-*SPTSSA* expression data sets. Under the MSigDB Collection's (c5.all.v7.1.symbols.gmt) enrichment analysis, the *SPTSSA* generated significant differences that were reported in the GSEA (*p* < 0.05). Our selection of the most highly enriched signaling pathways was based on their normalized enrichment scores (|*NES*| > 1) (Figure 4). The outcome revealed that negative regulation of response to oxidative stress, negative regulation of mitotic cell cycle, neuron death in response to oxidative stress, positive regulation of cellular catabolic process, and transcription factor complex were enriched in low expression phenotype (Figure 4(a)).

### 3.7. Connections between SPTSSA and TMB, MSI, and Immune Checkpoint

**Figure 5:**
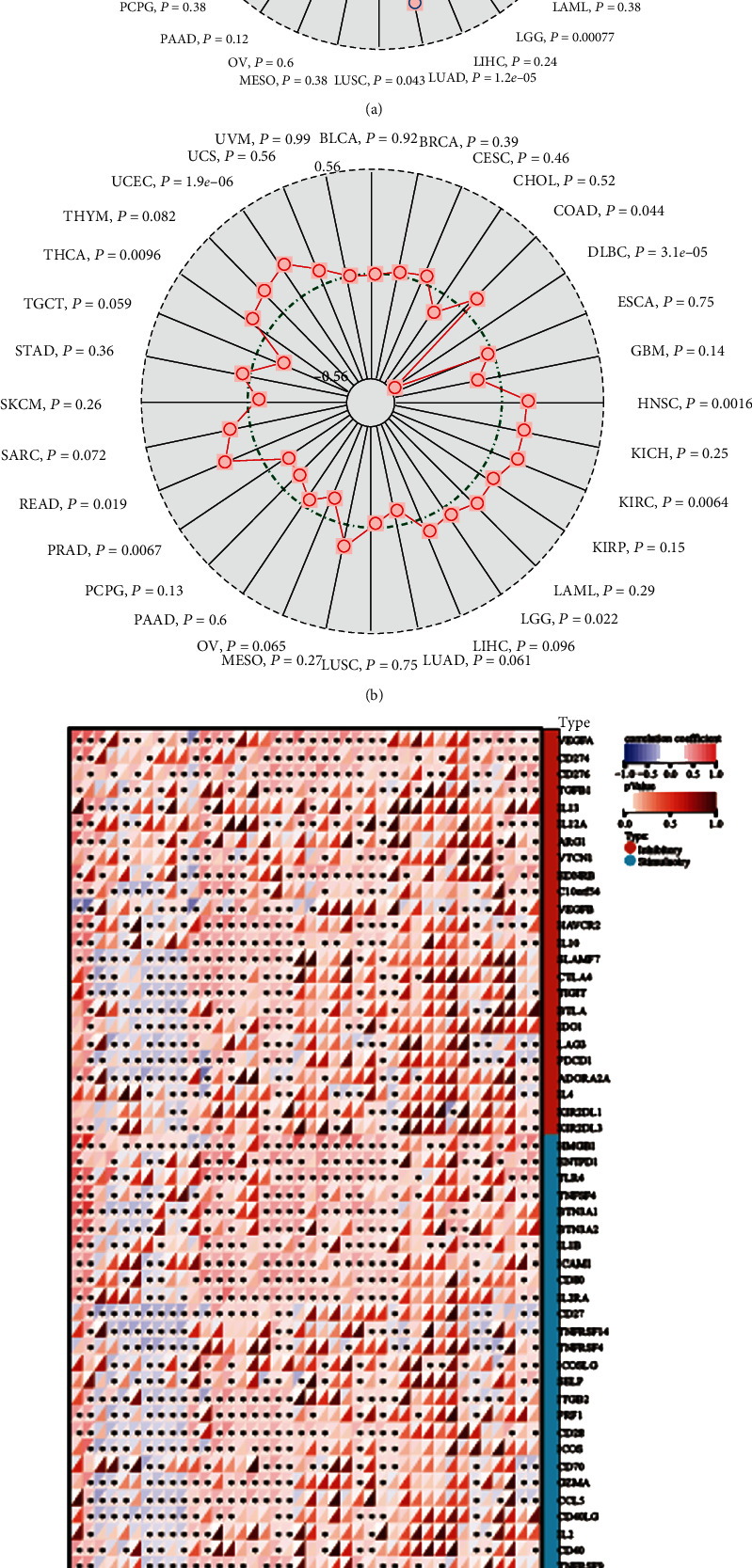
Correlations between SPTSSA and (a) TMB and (b) MSI. (c) Relationships between SPTSSA expression level and immune checkpoints.

There are studies which reveal that lower TMB values are associated with longer mean overall survival times, concluding that TMB is a marker of tumor malignancy [29]. Instability of microsatellites (MSI) results from mutations in DNA mismatch repair (MMR) genes, which fail to repair errors in DNA replication in repetitive sequences (microsatellites) [30]. There are studies found that MSI is associated with poor differentiation, proximal location, and failure of chemotherapy in colorectal cancers [31]. Thus, investigating the relationship between SPTSSA and TMB, MSI seem to be necessary. Regarding TMB, we found that SPTSSA expression level is correlated with TMB in GBM and LGG, while in MSI, we found no sense (Figures 5(a) and 5(b)).

Further, immune cells, checkpoint expression, and MSI status play a significant role in prognosis [32]. Thus, we further investigate the relationship between SPTSSA and immune checkpoint. And the results showed that SPTSSA correlated with most immune checkpoints (Figure 5(c)).

### 3.8. Associations between SPTSSA and DNA Methylation

DNA methylation is mediated by DNA methyltransferase (DNMT) and is affected by the environment [33–35]. Thus, we investigate the relationships between SPTSSA and four methytransferases (DNMT3B, DNMT3A, DNMT2, and DNMT1) (Supplementary Figure 5A). *p* < 0.05 and *R* > 0.20 indicated a significant and positive relationship, respectively. The results showed that SPTSSA has a positive correlation with four methytransferases in LGG, while in GBM, SPTSSA only has a positive correlation with DNMT3B, DNMT3A, and DNMT2.

### 3.9. Correlation of *SPTSSA* Expression and Infiltrating Immune Cells

**Figure 6:**
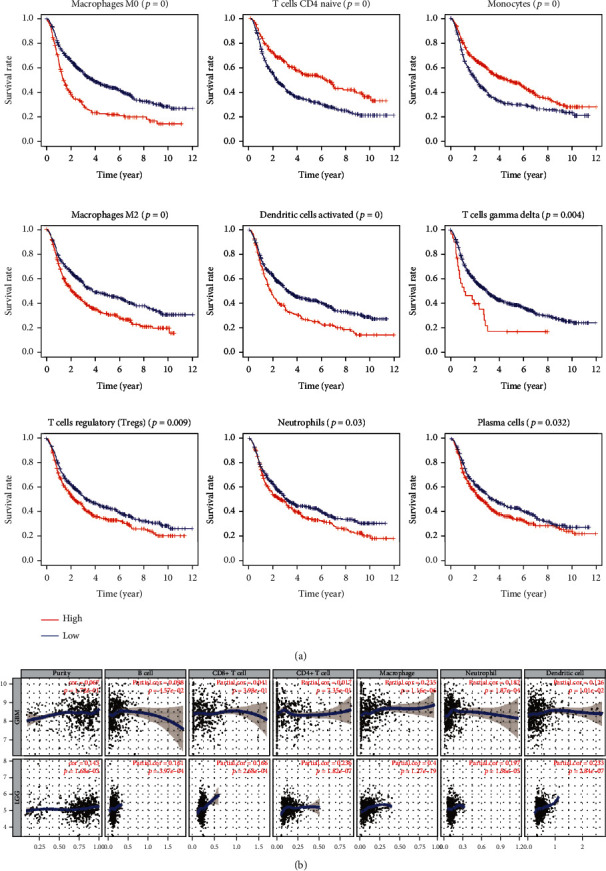
(a) Macrophage M0 cells (*p* < 0.001), T cells CD4 naive (*p* < 0.001), monocytes (*p* < 0.001), macrophages M2 (*p* < 0.001), dendritic cells activated (*p* < 0.001), T cells gamma delta (*p* = 0.004), T cells regulatory (Tregs) (*p* = 0.009), neutrophils (*p* = 0.030), and plasma cells (*p* = 0.032) were significantly associated with survival. (b) The correlation of immune cells and SPTSSA expression in the TIMER database.

We used CIBERSORT analysis to evaluate the correlation of *SPTSSA* expression with tumor-infiltrating immune cells (TIICs). As Figure 6(b) illustrates, our results demonstrated two statistically significant associations. First, the ratio of monocytes, NK cells (activated), T follicular helper (Tfh) cells, naive CD4+ T cells, memory B cells, and naive B cells were substantially lower in tumor cases with high *SPTSSA* expression. Second, eosinophils, dendritic cells (activated and resting), and macrophages (M0) were considerably higher in tumor cases with high *SPTSSA* expression.

### 3.10. Correlation of Infiltrating Immune Cells and Overall Survival

Using log-rank test survival analysis, we showed that high number of macrophage M0 cells (*p* < 0.001), T cells CD4 naive (*p* < 0.001), monocytes (*p* < 0.001), macrophages M2 (*p* < 0.001), dendritic cells activated (*p* < 0.001), T cells gamma delta (*p* = 0.004), T cells regulatory (Tregs) (*p* = 0.009), neutrophils (*p* = 0.030), and plasma cells (*p* = 0.032) were significantly associated with survival (Figure 6(a)).

### 3.11. Validation of Correlation between *SPTSSA* and Infiltrating Immune Cells

Using the TIMER database (Figure 6(b)), the correlation between the infiltrating immune cells and the *SPTSSA* expression was analyzed further to confirm our observation. The *SPTSSA* expression being significantly correlated with numbers of tumor-infiltrating dendritic cells, neutrophils, and macrophages was revealed clearly.

### 3.12. Immunohistochemistry (IHC)

**Figure 7:**
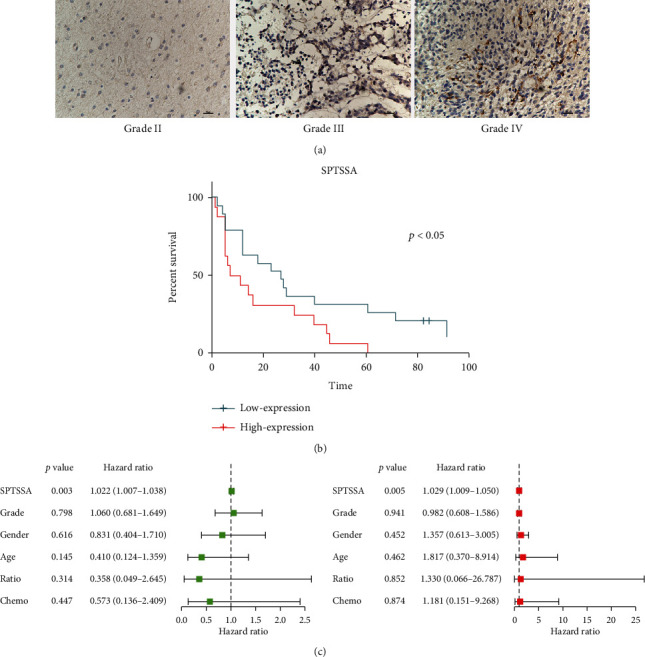
(a) IHC of grade II~IV glioma sample from the Affiliated Hospital of Nantong University. (a) Using TMA, grouped in median, high expression of SPTSSA predicts poor prognosis. (b, c) Univariate and multivariate Cox analyses indicated that SPTSSA was an independent predictor for OS.

To estimate the expression of *SPTSSA* in glioma tissues, we used IHC. Our findings revealed that in contrast to low-grade (I and II) gliomas, high-grade (III and IV) gliomas have highly expressed *SPTSSA* (Figure 7(a)). Log rank survival analysis indicated that high-*SPTSSA* expression in glioma was associated with poor survival (Logrank *p* = 0.045) (Figure 7(b)). In univariate analysis and multivariate analysis, *SPTSSA* is the only factor significantly associated with survival (Figure 7(c)).

### 3.13. Correlation Analysis between *SPTSSA* Expression and Immunosuppressive Markers

To investigate the relationship between *SPTSSA* and the tumor immunology infiltrating cells, we focused on the correlations between *SPTSSA* and well-known immunosuppression-related genes. Analysis with TMA showed that *SPTSSA* had highly positive correlation with CD8, CD66b, and CD20 (Supplementary Figure 5B).

## 4. Discussion

In the current study, by employing various bioinformatics analysis tools, we identified that *SPTSSA* expression was upregulated in diffuse glioma and associated with poor survival. We characterized *SPTSSA*-related biological processes and signaling pathways. Finally, we provided evidence that *SPTSSA* expression was correlated with tumor immune infiltrates by CIBERSORT, TIMER, IHC, and IF.

GEPIA is a web-based interactive tool for mining RNA sequencing data on TCGA and the GTEx databases [17, 18]. It covers over 45,000 genes. GEPIA allows experimental biologists with limited computational programming skills to perform large scale gene expression analyses [36, 37]. For current study, we utilized both differential expression analysis and patient survival analysis functions of GEPIA. We found that *SPTSSA* was not only differentially expressed in GBM but also associated with GBM survival. We further validated our findings by searching CGGA database.

*SPTSSA* is the gene encoding the small subunit A of serine palmitoyltransferase (SPT). It catalyzes the formation of sphingoid long-chain base backbone of sphingolipids [38]. Sphingolipids are structural compounds of biological membranes, and recent studies suggest sphingolipids can also serve as secondary messengers, participating in apoptosis, proliferation, senescence, angiogenesis, and vesicular trafficking [39, 40]. Because alterations in bioactive sphingolipids have been linked to cancer progression and prognosis, their key metabolic enzymes have been actively pursued as novel targets in cancer drug development [41–44]. The enrichment analysis showed that *SPTSSA* is related to the oxidative stress. Numerous studies have shown that the role of oxidative stress in glioma is quite important. To our best knowledge, this is the first study reporting the connection between *SPTSSA* expression, a key catalytic enzyme in sphingolipids synthesis, and cancer progression and prognosis.

To further shed light on the potential function of *SPTSSA* in GBM progression, we used both CIBERSORT and TIMER to correlate tumor-infiltrating immune cells with *SPTSSA* expression in GBM. Tumor-infiltrating immune cells are major member of the tumor microenvironment. They correlate with tumor prognosis and response to therapy. Traditionally, immunohistochemistry [45–47] and flow cytometry [48] are used to analyze and enumerate different subsets of immune cells. However, these methods are limited by the availability of markers and antibodies for identification of subtypes of immune cells as well as high quality tumor samples. CIBERSORT is a type of in silico tissue dissection method for enumerating different cell fractions from undissected tissue gene expression profiles through computational deconvolution analysis [20, 49]. Using pure immune cell subtype expression profiles, CIBERSORT can accurately estimate the immune cell subtypes of a tumor biopsy and enable the discovery of biomarkers and novel immunotherapeutic targets. Using CIBERSORT, we showed that the numbers of CD4 T memory cells and macrophage cells were positively correlated with *SPTSSA* expression, while the number of activated mast cells were negatively correlated with *SPTSSA* expression.

The TIMER was used to analyze the association with the survival of the tumor-infiltrating immune cells, to confirm our observations further. Cancer biologists are enabled in quantifying the abundance of tumor-infiltrating immune cells through a flexible and comprehensive mode by the TIMER (Tumor Immune Estimation Resource) [21]. The abundance of tumor-infiltrating immune cells from the bulk gene expression profiles are identified using the TIMER computational deconvolution methods. The association with overall survival was provided together with the association of the tumor-infiltrating immune cell abundance with the gene expression by TIMER. We successfully identified that higher numbers of activated mast cells were associated with better survival in GBM, that higher numbers of macrophage M0/M1 cells were associated with poor survival in GBM, and that there was a correlation with the *SPTSSA* expression of the tumor-infiltrating dendritic cells, neutrophils, and the macrophages. The *SPTSSA* being an independent prognosis factor and dysregulated in glioma was confirmed from the IF and the IHC results.

## 5. Conclusion

In summary, using bioinformatics tools, we identified high-*SPTSSA* expression in GBM tissues, and high-*SPTSSA* expression was associated with poor survival. In silico tumor-infiltrating immune cell analysis suggests that high-*SPTSSA* expression was associated with high number of specific subtype immune cells. Future experimental studies are needed to explore the potential of *SPTSSA* as prognostic marker as well as novel immunotherapy target for GBM.

## Figures and Tables

**Table 1:** Clinical information analysis based on CGGA database.

		Total (749)	Low expression (374)	High expression (375)	*χ* ^2^	*p*
PRS_type	Primary	502	267	235	6.691	0.035
	Recurrent	222	95	127		
	Secondary	25	12	13		

Grade	WHO II	218	127	91	16.663	0
	WHO III	240	128	112		
	WHO IV	291	119	172		

Gender	Male	307	167	140	4.147	0.042
	Female	442	207	235		

Age	≤41	342	169	173	0.068	0.795
	>41	407	205	202		

Radio_status	No	124	74	50	5.644	0.018
	Yes	625	300	325		

Chemo_status	No	229	134	95	9.718	0.002
	Yes	520	240	280		

IDH_mutation_status	Wildtype	339	157	182	3.247	0.072
	Mutant	410	217	193		

1p19q_codeletion_status	Noncodel	614	283	331	7.888	0.005
	Codel	155	91	64		

## Data Availability

The datasets generated during and/or analyses during the current study are available in the Chinese Glioma Genome Atlas Network (CGGA).

## Abbreviations

*SPTSSA*: The small subunit A of serine palmitoyltransferase
GEPIA: Gene Expression Profiling Interactive Analysis
CGGA: The Chinese Glioma Genome Atlas
TCGA: The Cancer Genome Atlas
GSEA: Gene Set Enrichment Analysis
TIMER: Tumor Immune Estimation Resource
IHC: Immunohistochemistry
GBM: Glioblastoma multiforme
TAMs: Tumor-associated macrophages
TINs: Tumor-infiltrating neutrophils
DEGs: Differentially expressed genes
GSEA: Gene Set Enrichment Analysis
NES: Normalized enrichment score
TMA: Tissue microarray
IF: Immunofluorescence.

## Supplementary Materials

Supplementary 1 Supplementary Figure 1: (A) a nomogram showing SPTSSA and different clinical characteristics based on TCGA database. (B) Calibration curve of nomogram.

Supplementary 2 Supplementary Figure 2: Cox analysis of SPTSSA between (A) OS, (B) PFI, (C) DSS, and (D) DFI.

Supplementary 3 Supplementary Figure 3: (A) the immunotherapy response between SPTSSA expression level and anti-PD-1/CTLA4. (B) AUC curve of SPTSSA expression level in anti-PD-1/CTLA4 immunotherapy. (C) Relationships between SPTSSA and immune suppressive factors, immune promoting factors, MHC factors, chemokine, and receptors.

Supplementary 4 Supplementary Figure 4: connections between *SPTSSA* and genomic alteration in (A) GBM and (B) LGG.

Supplementary 5 Supplementary Figure 5: (A) correlations between SPTSSA and DNA methyltransferase. (B) The analysis of glioma tumor-infiltrating lymphocytes (TILs) with different SPTSSA expression.
