# Supplementary figures and images for: SPTSSA Is a Prognostic Marker for Glioblastoma Associated with Tumor-Infiltrating Immune Cells and Oxidative Stress
Source: Oxid Med Cell Longev. 2022 Aug 24;2022:6711085. doi: 10.1155/2022/6711085 (PMC9434331; doi:10.1155/2022/6711085)

**A**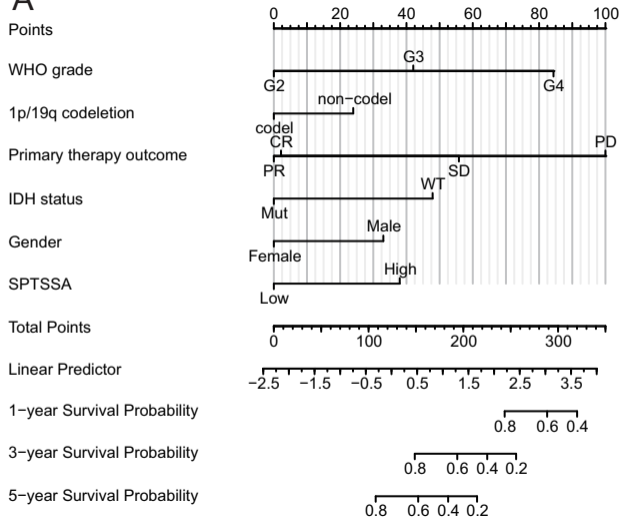**B**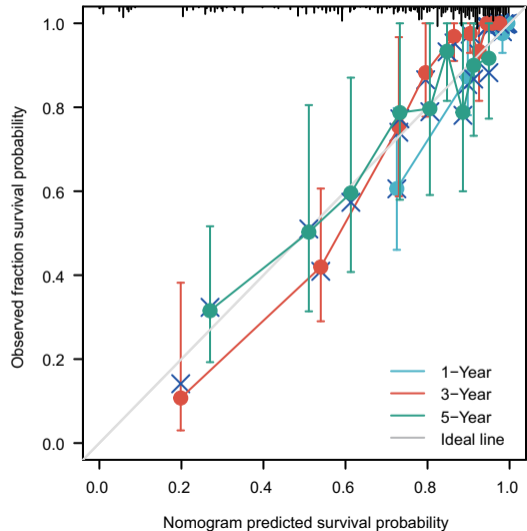

Supplement: Supplementary 1 — Supplementary Figure 1: (A) a nomogram showing SPTSSA and different clinical characteristics based on TCGA database. (B) Calibration curve of nomogram. [file 6711085.f1.pdf]

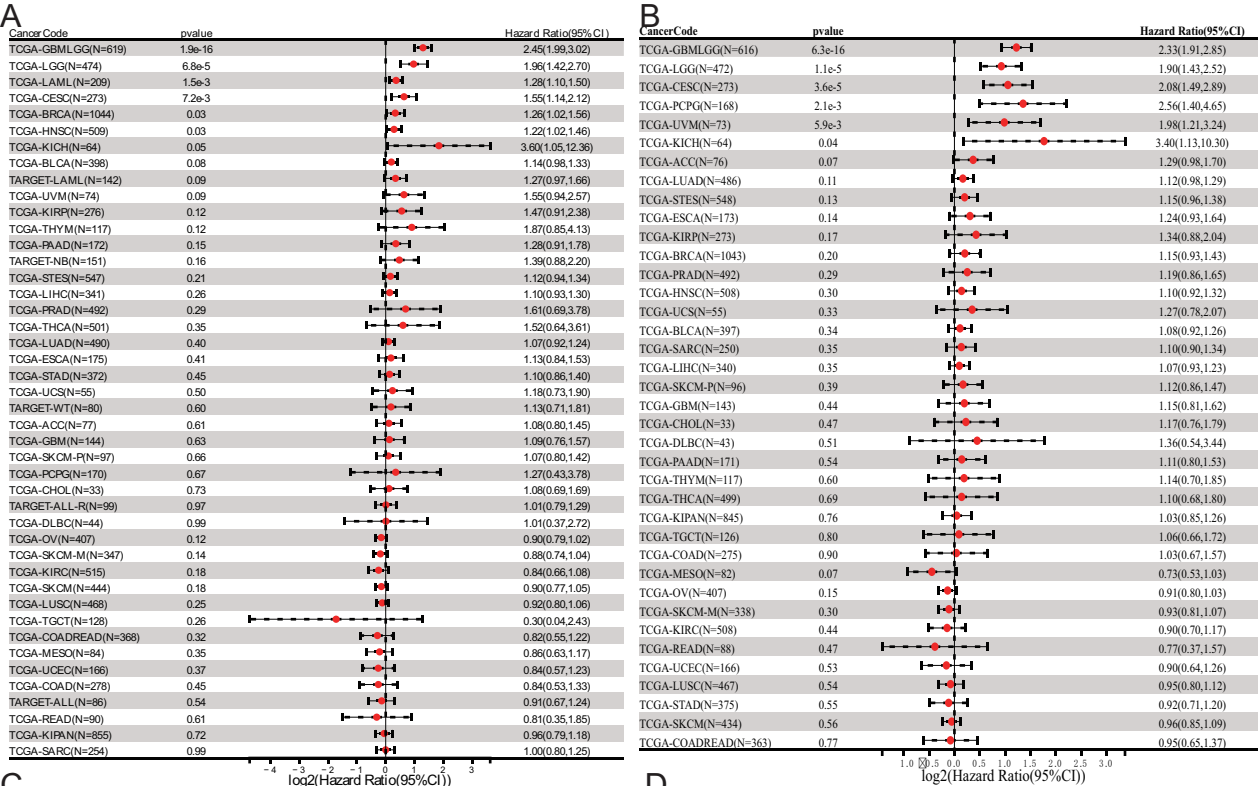

Supplement: Supplementary 2 — Supplementary Figure 2: Cox analysis of SPTSSA between (A) OS, (B) PFI, (C) DSS, and (D) DFI. [file 6711085.f2.pdf]

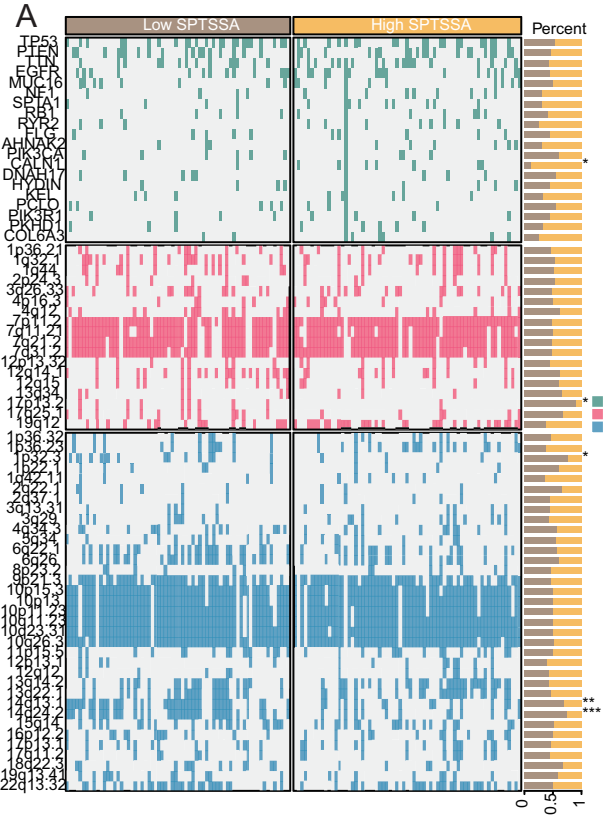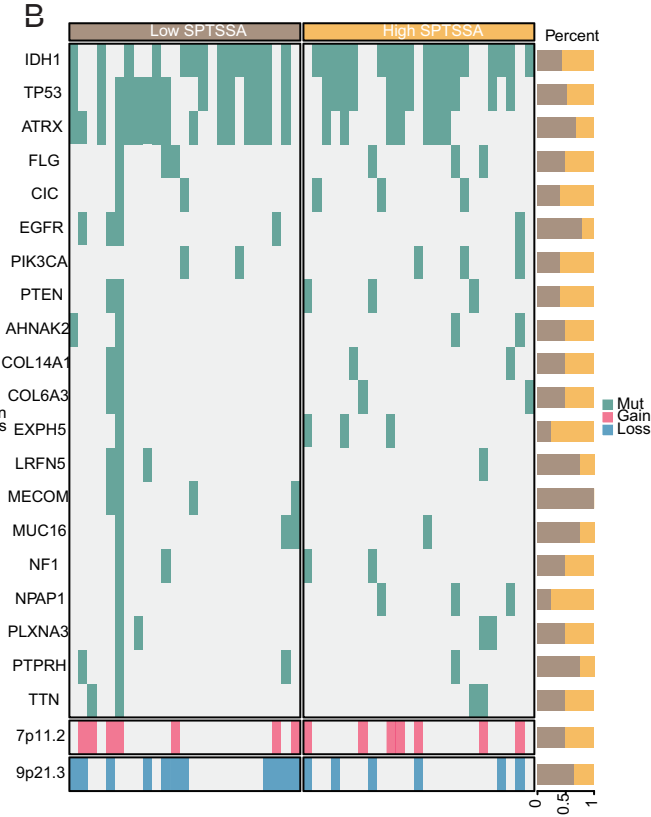

Supplement: Supplementary 4 — Supplementary Figure 4: connections between SPTSSA and genomic alteration in (A) GBM and (B) LGG. [file 6711085.f4.pdf]
