# Supplementary material for: SPTSSA Is a Prognostic Marker for Glioblastoma Associated with Tumor-Infiltrating Immune Cells and Oxidative Stress
Source: Oxid Med Cell Longev. 2022 Aug 24;2022:6711085. doi: 10.1155/2022/6711085 (PMC9434331; doi:10.1155/2022/6711085)
Supplement: Supplementary 3 — Supplementary Figure 3: (A) the immunotherapy response between SPTSSA expression level and anti-PD-1/CTLA4. (B) AUC curve of SPTSSA expression level in anti-PD-1/CTLA4 immunotherapy. (C) Relationships between SPTSSA and immune suppressive factors, immune promoting factors, MHC factors, chemokine, and receptors. [file 6711085.f3.pdf]

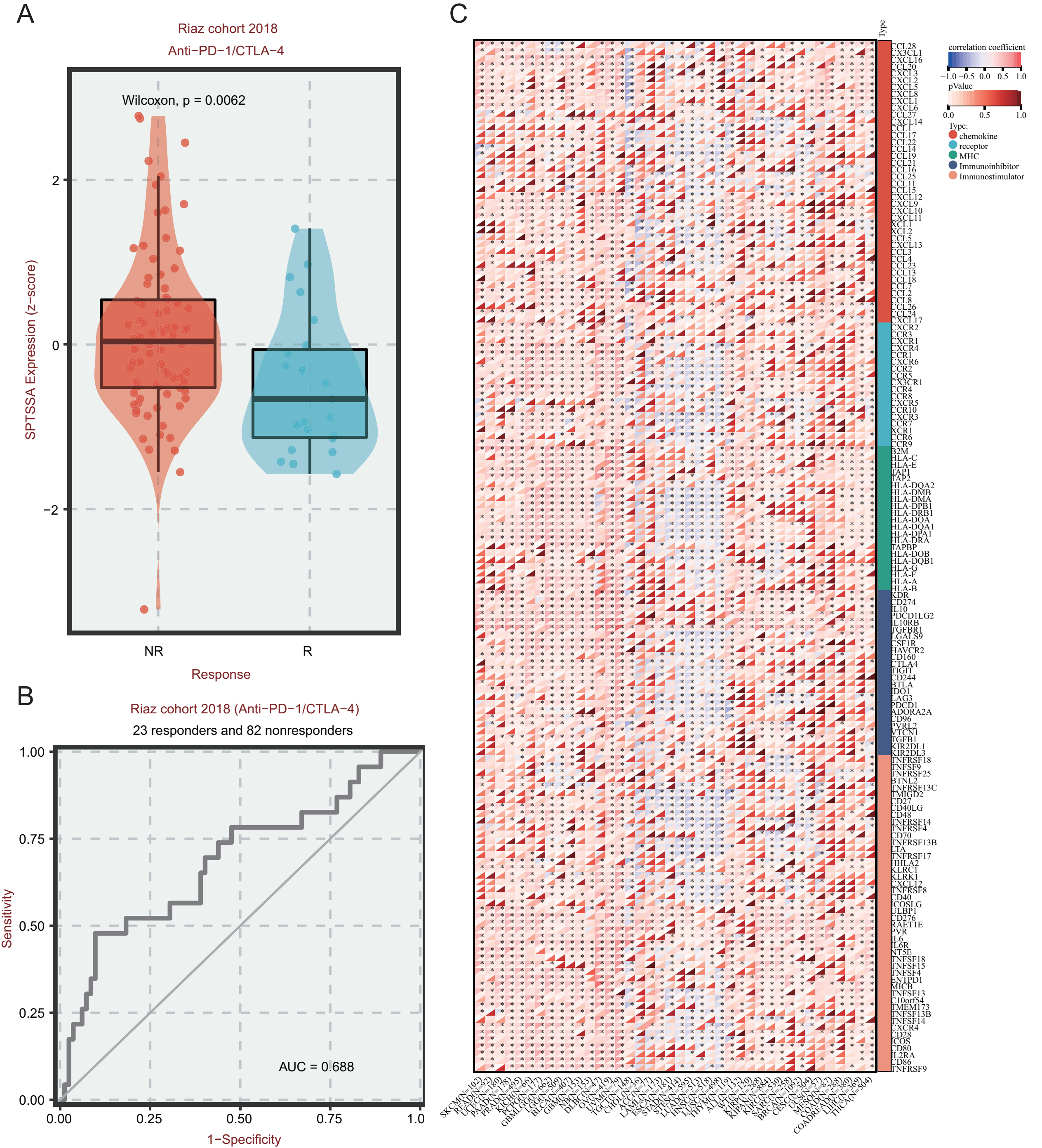
A
Riaz cohort 2018
Anti−PD−1/CTLA−4
Wilcoxon, p = 0.0062
SPTSSA Expression (z−score)
2
0
−2
NR
R
Response
B
Riaz cohort 2018 (Anti−PD−1/CTLA−4)
23 responders and 82 nonresponders
Sensitivity
1.00
0.75
0.50
0.25
0.00
AUC = 0.688
0.00
0.25
0.50
0.75
1.0
1−Specificity
C
Type
correlation coefficient
−1.0 −0.5 0.0 0.5 1.0
pValue
0.0 0.5 1.0
Type:
chemokine
receptor
MHC
Immunoinhibitor
Immunostimulator
CCL28
CX3CL1
CXCL16
CCL20
CXCL3
CXCL2
CXCL5
CXCL8
CXCL1
CXCL6
CCL27
CXCL14
CCL1
CCL17
CCL22
CCL14
CCL19
CCL21
CCL16
CCL25
CCL11
CCL15
CXCL12
CXCL9
CXCL10
CXCL11
XCL1
XCL2
CCL5
CXCL13
CCL3
CCL4
CCL23
CCL13
CCL18
CCL7
CCL2
CCL8
CCL26
CCL24
CXCL17
CXCR2
CCR3
CXCR1
CXCR4
CCR1
CXCR6
CCR2
CCR5
CX3CR1
CCR4
CCR8
CXCR5
CCR10
CXCR3
CCR7
XCR1
CCR6
CCR9
B2M
HLA-C
HLA-E
TAP1
TAP2
HLA-DQA2
HLA-DMB
HLA-DMA
HLA-DPB1
HLA-DRB1
HLA-DOA
HLA-DQA1
HLA-DPA1
HLA-DRA
TAPBP
HLA-DOB
HLA-DQB1
HLA-G
HLA-F
HLA-A
HLA-B
KDR
CD274
IL10
PDCD1LG2
IL10RB
TGFBR1
LGALS9
CSF1R
HAVCR2
CD160
CTLA4
TIGIT
CD244
BTLA
IDO1
LAG3
PDCD1
ADORA2A
CD96
PVRL2
VTCN1
TGFB1
KIR2DL1
KIR2DL3
TNFRSF18
TNFSF9
TNFRSF25
BTNL2
TNFRSF13C
TMIGD2
CD27
CD40LG
CD48
TNFRSF14
TNFRSF4
CD70
TNFRSF13B
LTA
TNFRSF17
HHLA2
KLRC1
KLRK1
CXCL12
TNFRSF8
CD40
ICOSLG
ULBP1
CD276
RAET1E
PVR
IL6
IL6R
NT5E
TNFSF18
TNFSF15
TNFSF4
ENTPD1
MICB
TNFSF13
C10orf54
TMEM173
TNFSF13B
TNFSF14
CXCR4
CD28
ICOS
CD80
IL2RA
CD86
TNFRSF9
