# Supplementary material for: SPTSSA Is a Prognostic Marker for Glioblastoma Associated with Tumor-Infiltrating Immune Cells and Oxidative Stress
Source: Oxid Med Cell Longev. 2022 Aug 24;2022:6711085. doi: 10.1155/2022/6711085 (PMC9434331; doi:10.1155/2022/6711085)
Supplement: Supplementary 5 — Supplementary Figure 5: (A) correlations between SPTSSA and DNA methyltransferase. (B) The analysis of glioma tumor-infiltrating lymphocytes (TILs) with different SPTSSA expression. [file 6711085.f5.pdf]

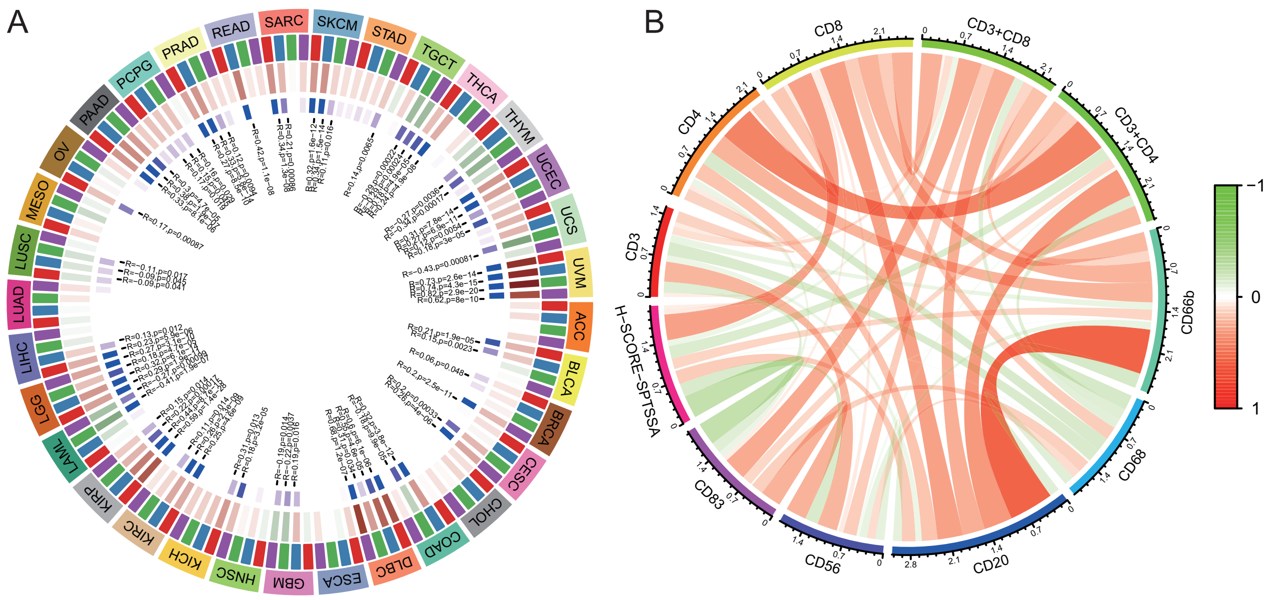
A
B
SARC
SKCM
STAD
TGCT
THCA
THYM
UCEC
UCS
UVM
ACC
BLCA
BRCA
CESC
CHOL
COAD
DLBC
ESCA
GBM
HNSC
KICH
KIRC
KIRP
LAML
LGG
LIHC
LUAD
LUSC
MESO
OV
PAAD
PCPG
PRAD
READ
CD8
CD3+CD8
CD3+CD4
CD66b
CD68
CD20
CD56
CD83
H-SCORE-SPTSSA
CD3
CD4
